# Barriers, Facilitators, and a Proposed Model of Care for Implementation of Upper Limb Distributed Practice Approaches for Children with Unilateral Cerebral Palsy

**DOI:** 10.3390/jcm14030924

**Published:** 2025-01-30

**Authors:** Emma Taylor, Susan Greaves, Brian Hoare

**Affiliations:** 1 Cerebral Palsy Group, 74 Faraday Street, Carlton, VIC 3053, Australia; emma@tayloredconsult.com (E.T.); sue.greaves1@outlook.com (S.G.); 2 School of Allied Health, Australian Catholic University, 115 Victoria Parade, Fitzroy, VIC 3065, Australia; 3 Discipline of Occupational Therapy, La Trobe University, Plenty Road, Bundoora, VIC 3086, Australia; 4 Department of Paediatrics, Monash University, Clayton VIC 3800, Australia

**Keywords:** cerebral palsy, occupational therapy, bimanual therapy, constraint-induced movement therapy, implementation

## Abstract

**Background/Objectives**: There is high-level research evidence supporting task-focused upper limb therapy models for children with unilateral cerebral palsy (CP). However, a knowledge gap exists in understanding how to effectively implement distributed practice approaches in clinical practice and the effect on the development of bimanual performance. This study aims to evaluate clinical outcomes, examine key considerations for implementation outcomes, and propose a Model of Care for children with unilateral CP. **Methods**: A mixed-methods approach was applied, including a retrospective case series with an observational descriptive design. A convenience sample of nine children (<5 years of age) with unilateral CP who received multiple blocks of distributed, evidence-based upper limb therapy approaches between 2014 and 2020 were included. Outcomes were evaluated using the Assisting Hand Assessment family of assessments. A Model of Care framework was informed by the Updated Consolidated Framework for Implementation Research and the Conceptual Model for Implementation Research. **Results**: A total of 59 blocks of upper limb therapy (10 mCIMT and 49 bimanual therapy) were delivered, ranging from two to nine blocks (mean = 6.6) for each child. All children demonstrated improved outcomes in bimanual performance with an average change of 14 AHA units (range 1–22). Barriers to implementation included complexity and cost. Facilitators included the evidence base and adaptability of the approaches that allowed clinicians to respond to an individual child and family’s needs. Informed by evidence-based protocols and visual analysis of data, and in consideration of the barriers and/or facilitators to implementation from this study, a Model of Care for implementation of upper limb distributed practice approaches for children with unilateral CP in clinical practice is proposed. **Conclusions**: Implementing repeated, distributed blocks of evidence-based upper limb therapy in a clinical setting for children with unilateral CP led to incremental improvements in bimanual performance. There are a range of barriers and facilitators to the implementation of distributed practice approaches in clinical practice. The Model of Care outlines best practice care and service delivery for children with unilateral CP and their families and aims to support clinical practice and the future examination of implementation-effectiveness in practice.

## 1. Introduction

Unilateral cerebral palsy (CP) is the most common form of CP in Australia [1]. For children with unilateral CP, impairment of the more-affected arm and hand leads to difficulties with early skill development, such as reaching, holding, and grasping objects. The reduced unimanual function also creates difficulty with learning how to use two hands together for bimanual play [2]. All children with unilateral CP will increase their ability to use their more-affected upper limb in bimanual activities over time. However, the individual rate of improvement and ability limit can be vastly different [3,4,5]. While the specific reasons for this variability remain unknown, specific factors that may influence the development of bimanual performance include the location, timing, and type of brain impairment, gestational age, gender, presence of seizure activity, cognitive/perceptual impairment, the type/severity of the movement disorder, and type and timing of therapy services received, along with social/cultural/environmental factors [5,6,7]. For children with CP, harnessing the mechanisms of activity or experience-dependent neural plasticity [8,9] through early therapy is critical [10], as the most rapid gains in bimanual abilities occur in the first 3 years of life [3,11,12].

Contemporary models of evidence-based upper limb therapy for children with unilateral CP, such as constraint-induced movement therapy (CIMT) and bimanual therapy, are based on motor learning theory and involve the delivery of time-limited, goal-directed, intensive blocks of practice [13]. There are, however, broad variations in the dosage, content, and mode of delivery across the different therapy approaches that have been developed [14]. *Intensive* therapy approaches are implemented using a high duration and frequency of sessions over a short period, such as 6 h/day, 5 times per week for 2 weeks. These dosage parameters have been found to be an effective way to deliver CIMT [15] and bimanual therapy approaches, such as Hand Arm Bimanual Intensive Training (HABIT) [16]. Bimanual therapy and modified CIMT (mCIMT) have also been applied as a *distributed* approach where practice is implemented using a lower duration and frequency of sessions over a longer period (such as 8 weeks) to optimize learning outcomes [17,18,19]. A key component of a distributed therapy approach is the use of home programs, which ensures that consistent practice occurs during the defined blocks of therapy, providing the repetition needed to meet dosage requirements [20,21]. Research examining distribution-of-practice effects outside the CP population suggests that optimal learning outcomes are achieved when practice is distributed over time [22,23]; however, there is an emerging trend for families of children with CP to seek massed practice, high-dose therapy approaches [24].

While intensive and distributed upper limb activity-level therapy approaches for children with unilateral CP aged over 18 months are supported by high-level evidence [25,26], not all children respond to a single block of therapy [27]. This is not surprising, as these therapy approaches were not intended to be applied as a one-off. While there are some promising outcomes for the cumulative effect of repeating blocks of therapy [28,29,30,31], there is limited evidence about the long-term effects of implementing distributed therapy approaches across early childhood in clinical practice [32]. Additional research is required to determine whether children may respond to multiple blocks of therapy and whether each block adds an incremental benefit or whether the gains are one-off, short-term, or plateau over time [33].

The aim of this study is to describe the clinical outcomes from the implementation of multiple distributed models of upper limb therapies in a cohort of children with unilateral CP and to reflect on the key implementation strategies and outcomes observed (retrospectively) in a pragmatic clinical setting. Measuring the cumulative effect of multiple blocks of upper limb therapy from early infancy into early childhood is difficult. Distinguishing the effect of therapy from those changes that occur naturally during development or from other therapy adds to these challenges [34]. The only way to eliminate all possibility of a confounding variable is via a prospective randomized controlled study [35] However, a randomized controlled trial methodology is not possible or appropriate for a study that aims to evaluate longitudinal outcomes for children with unilateral CP in a pragmatic clinical setting. The use of an implementation science research framework in this study provides a dual focus that outlines both an evaluation of intervention effectiveness (the “what” you do) and the extent and quality of the implementation strategies being used (the “how” you do it) to ensure the effective adoption, implementation, or sustainment of the interventions [36]. Guided by the implementation strategies, a Model of Care will also be proposed to support clinical practice and the future examination of implementation effectiveness in practice.

## 2. Methods

### 2.1. Participants

A convenience sample of 9 infants and preschool-aged children (<5 years) with unilateral CP who attended a specialist pediatric private practice (CPTherapy) in Melbourne, Australia between 2014 and 2020 was included. To minimize selection bias, families of eligible children were invited to participate by email and/or phone by an investigator with no relationship to the child and family (ET). Written informed consent was obtained prior to enrolment in the study. Inclusion criteria included having a diagnosis of unilateral CP documented by a medical professional, <5 years of age on commencement of services, received > one block of either mCIMT or bimanual therapy during the study period, and had bimanual performance data collected pre/post each block of upper limb intervention.

### 2.2. Study Design and Setting

#### 2.2.1. Phase One

Phase One of this study used a retrospective case series with an observational descriptive design. It was approved by the Human Research Ethics Committee of Monash Health, Victoria, Australia (HREC 74087; RES-21-0000310L). Descriptive data were obtained from families at the initial assessment. Due to age at study entry, children’s manual ability was classified using the Manual Ability Classification System (MACS) [37] at the conclusion of the study period. The Mini-MACS was unavailable at the time of the study period. Each child was assessed using either the Hand Assessment for Infants (HAI) [38], the Mini-Assisting Hand Assessment (Mini-AHA) [39], or the Assisting Hand Assessment (AHA) [40]. These assessments are conducted by video recording the infant or child participating in a structured 10 to 15 min play session using toys from a test kit and are then scored according to assessment criteria. The specific assessment used was guided by the age of the child. Where possible, assessments were repeated after each block to evaluate the effect of therapy. In some cases, the post-intervention assessment served as the baseline assessment for the next block. This reflects the pragmatic nature of this study evaluating clinical practice. The assessment was non-blinded; however, each film was coded and scored by a single assessor in random order.

#### 2.2.2. Phase Two

**Figure 1:**
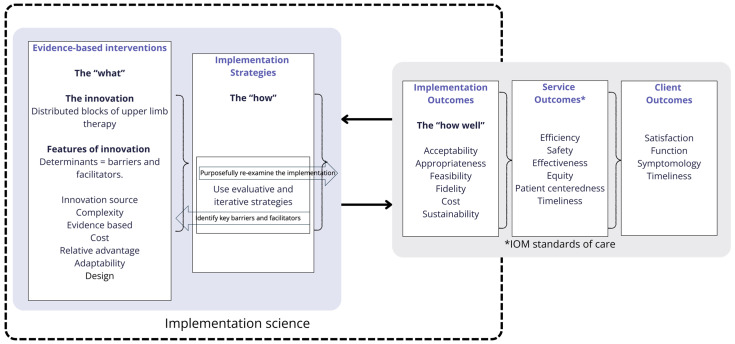
Adapted Conceptual Model for Implementation Research (CFIR).

For Phase Two of this study, the Updated Consolidated Framework for Implementation Research [41] and the Conceptual Model for Implementation Research (CFIR) [42] were used to guide a retrospective review to identify key implementation strategies and outcomes to consider in the development of a proposed Model of Care. These are research frameworks from the field of implementation science. The updated CFIR is a determinant framework with five domains, including characteristics of the intervention (the “innovation”), outer setting, inner setting, characteristics of the individuals involved, and the process of implementation. These domains include constructs that incorporate contextual factors that may impact implementation effectiveness [41]. Determinants are independent variables that can be described as barriers and/or facilitators to the successful implementation of the innovation, and understanding what they are is an important implementation strategy [43]. This study describes the use of an “innovation” (or the “thing” that is being implemented) as multiple blocks of distributed, evidence-based upper limb therapy as described in the TIDieR-Rehab checklist (see Supplementary Table S1) [44]. The authors retrospectively identified key characteristics of the “innovation” that may be considered a barrier and/or facilitator to implementation. These can include the Adaptability of the innovation—can it be modified, tailored, or refined to fit local context or needs; the Evidence base—is there robust evidence supporting its effectiveness; the Design—how well is it designed or presented; and the Complexity—is the innovation complex in scope and nature [41]. This process was guided by Proctor et al.’s (2009) [42] conceptual model of implementation research (see Figure 1), which highlights the interaction between delivering an evidence-based intervention (the what), using effective implementation strategies to do it (the how), and then evaluating the outcomes of implementing the intervention (how well). As described using the Expert Recommendations for Implementing Change (ERIC) taxonomy [43], examples of implementation strategies include ensuring the use of evaluative and iterative strategies, such as identifying and understanding barriers and facilitators before, during, and while service provision occurs, and incorporating strategies to evaluate and purposefully re-examine the implementation to determine if the outcomes of the evidence-based intervention are as intended [43]. In this study, the authors retrospectively identified implementation outcomes monitored in the clinical setting during the study period, which most directly impacted the provider and participant (parent) adherence to upper limb therapy protocols. These included acceptability, appropriateness, fidelity, feasibility, cost, and sustainability [42].

Following the identification and consideration of the implementation strategies and outcomes, Phase 2 of this study used a methodology for the Model of Care framework [45] to propose a clinical Model of Care for the implementation of multiple distributed models of upper limb therapy. The process of designing a Model of Care involves identifying challenges in service delivery, designing and documenting solutions, supporting the health system to implement the new ideas, and evaluating outcomes of the Model of Care [45]. A Model of Care used in conjunction with reporting guidelines, e.g., TIDieR, aims to incorporate the key features and unique principles of a specific healthcare service within a framework that can facilitate the implementation and concurrent evaluation of the care [46].

### 2.3. Data Analysis

#### 2.3.1. Case Series Data Review (Phase One)

Data from the Mini-AHA and AHA for each child over time were plotted graphically for visual analysis. Along with the inspection of descriptive statistics, the visual analysis of data was considered the most appropriate method for this study [47]. No quantitative statistical analyses were undertaken. While each measure produces a total score on a 0 to 100 interval-level scale, i.e., Mini-AHA units or AHA units, the relationship between the scores from each of these measures is currently unknown and, therefore, plotted separately.

#### 2.3.2. Identification of Implementation Strategies and Outcomes (Phase Two)

A retrospective review was completed by the authors to identify key implementation strategies used and outcomes evaluated during the study period (see Figure 1). This included identifying characteristics of the “innovation” as barriers and/or facilitators to implementation and examining implementation outcomes influencing adherence to upper limb therapy protocols. How well the innovation was being implemented in the pragmatic clinical setting was monitored through direct observation by the clinician of the child’s performance in therapy, changes in bimanual performance pre/post-intervention, and provider/parent communication. The development of the Model of Care was informed by the evidence-based protocols for upper limb therapy, visual analysis of data from case series participants, and a review of the barriers and/or facilitators to implementation of the interventions across the study period.

## 3. Results

### 3.1. Descriptive Data

Nine children with unilateral CP were included (six males; six right side more affected; mean age at first contact with CPTherapy was 11.6 months (range 6 to 33 months)); MACS I (*n* = 2), MACS II (*n* = 7) (see Table 1).

### 3.2. Case Series Data Review (Phase One)

**Figure 2:**
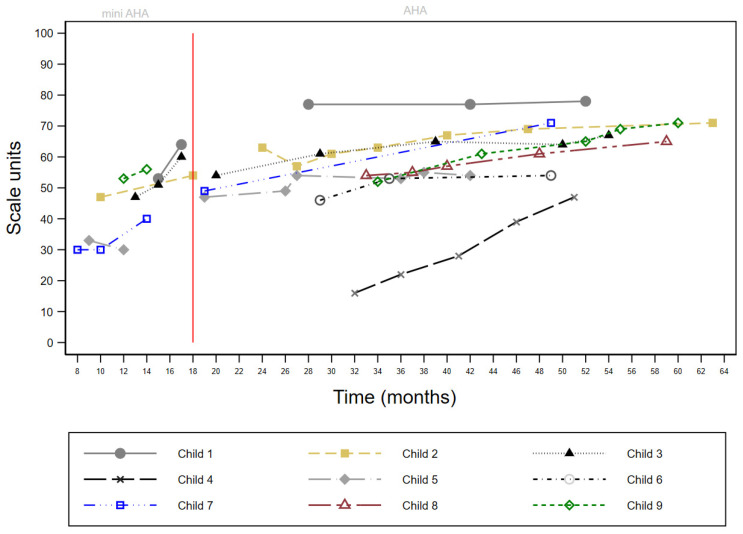
Change in Mini-Assisting Hand Assessment (pre-18 months) and Assisting Hand Assessment scores.

Data from the HAI were limited to four children due to the more recent development of this test. Mini-AHA data were available for seven children who first accessed services before 18 months of age. Of these seven children, six had repeated assessments and demonstrated an average change of 6.8 Mini-AHA units (range −3–13) (see Figure 2). One child had a single Mini-AHA assessment timepoint. All children had repeated AHA assessments from 18 months to 5 years, with an average change of 14 AHA units (range 1–22) (see Figure 2). A total of 59 blocks of upper limb therapy (10 mCIMT and 49 bimanual therapy) were delivered, ranging from two to nine blocks (mean = 6.6) for each child. In the first 2 years of therapy, each child received an average of three blocks per year. This decreased to two blocks per year before transitioning to GDT. The time between blocks was shorter for initial blocks (block 1 to block 2, mean = 1.4 months) and increased for later blocks (block 4 to block 5, mean = 3.1 months). Five children transitioned to GDT during the study period (mean age at transition: 3 years, 3 months). The children with higher baseline AHA scores transitioned to GDT at an earlier age. For most children who transitioned to GDT, bimanual performance continued to improve as measured using the AHA. During the study period, three children moved to a different region, and one child underwent a hemispherectomy for the management of epilepsy at 4 years of age and was referred to a service that was able to provide home-based services.

### 3.3. Identification of Implementation Strategies and Outcomes (Phase Two)

The barriers and facilitators to the implementation of the evidence-based upper limb therapy approaches (the innovation) were retrospectively identified during the study period. Additionally, strategies to purposefully examine the implementation of the innovation were also identified.

#### 3.3.1. Barriers

Barriers to the implementation of multiple distributed blocks of evidence-based upper limb therapy include the complexity of the interventions. Due to the contemporary nature of the models of therapy used in this study, clinicians required high-level knowledge and skills in the delivery of these models of therapy. Typically, this would require specific training for each approach; however, both clinicians in this study were involved in the development of some of the approaches used, and/or they teach other clinicians about the implementation of these approaches in clinical practice. Weekly sessions were provided during blocks of therapy to ensure adequate, consistent support and coaching, and education was provided to parents/caregivers. This included education about the key ingredients for the specific therapy approaches. Effective education also required therapists to have high-level knowledge about the range of motor learning strategies that can be applied to optimize skill acquisition, generalization, and transference. Ongoing coaching was required to continually re-evaluate goals and maximize the effectiveness of the limited periods that parents/caregivers have in their daily routine to implement the home program. It included how to make structured home-based play sessions fun, engaging, and meaningful.

In addition to the complexity, a barrier to implementation included the cost. Regular blocks of therapy, where a child is seen once per week, require ongoing funding. Both CIMT and bimanual therapy distributed practice approaches also require the commitment of the parent/caregiver to complete daily home practice programs to ensure dosage requirements are met. They require significant resources, including access to a variety of toys and equipment (e.g., appropriate seating, table). For infants and children receiving CIMT, the use of a constraint device (a mitt) was required. This needed to be customized and fitted for each child. For clinicians, there were also substantial costs. To administer and score the measures used to objectively evaluate individual outcomes and plan future therapy sessions (HAI, Mini-AHA, AHA), clinicians needed to attend certification courses. In addition, there were costs associated with access to CPToys, the purchase of therapy products such as toys and equipment, and the ongoing costs for maintaining a clinic space and clinical service.

#### 3.3.2. Facilitators

Facilitators to the implementation of multiple distributed blocks of evidence-based upper limb therapy include the evidence base of the intervention, which is extensive and well established. There is a significant relative advantage to using CIMT and bimanual therapy compared with usual care [26]. The adaptability of the innovation allowed clinicians to respond to an individual child and family’s needs. This included individualized goal setting, selection of the specific therapy approach, the length of each block (6 to 8 weeks), the number of blocks provided, and the length of time between blocks (period of consolidation). A further facilitator includes the design, with clearly defined blocks of intervention. The use of online resources, such as CPToys [48], helped support parents’/caregivers’ understanding of their child’s specific action-focused goals and matched toys to these goals to support home practice. The parents’ ability to implement the home program and facilitate the desired goal-related skills and actions was facilitated through weekly coaching, education, and discussion. Goals were reviewed weekly and adapted in line with a child’s skill acquisition. For children receiving CIMT, a logbook provided a written record of the dosage of practice applied in the home environment and helped to facilitate an understanding of the importance of dosage of practice for this model of therapy.

#### 3.3.3. Implementation Outcomes

The implementation outcomes monitored in this study included acceptability and appropriateness, fidelity, feasibility, cost, and sustainability. The parents/caregivers and children found the models of intervention to be acceptable, including the use of a restraint mitt for the implementation of the CIMT approaches. All children and their families engaged in the scheduled blocks of therapy and use of home programs throughout the study period. The perceived fit of the innovation to match parent goals and the child’s needs was aligned. Due to the small number and expertise of the therapists providing the innovation (*n* = 2), the fidelity of the innovation was strong. Clinicians and parents/caregivers adhered to the approach being applied, as the key ingredients of each approach were clearly identified and articulated during blocks of therapy, and the dosage of practice was specified and monitored. For children receiving CIMT, a logbook provided a written record of the dosage of practice applied in the home environment. The innovation demonstrated feasibility with families engaging in multiple, repeated blocks of therapy, which were successfully carried out in the clinic and home-based settings [49].

### 3.4. Development of a Proposed Model of Care (MOC) for Upper Limb Therapy

**Figure 3:**
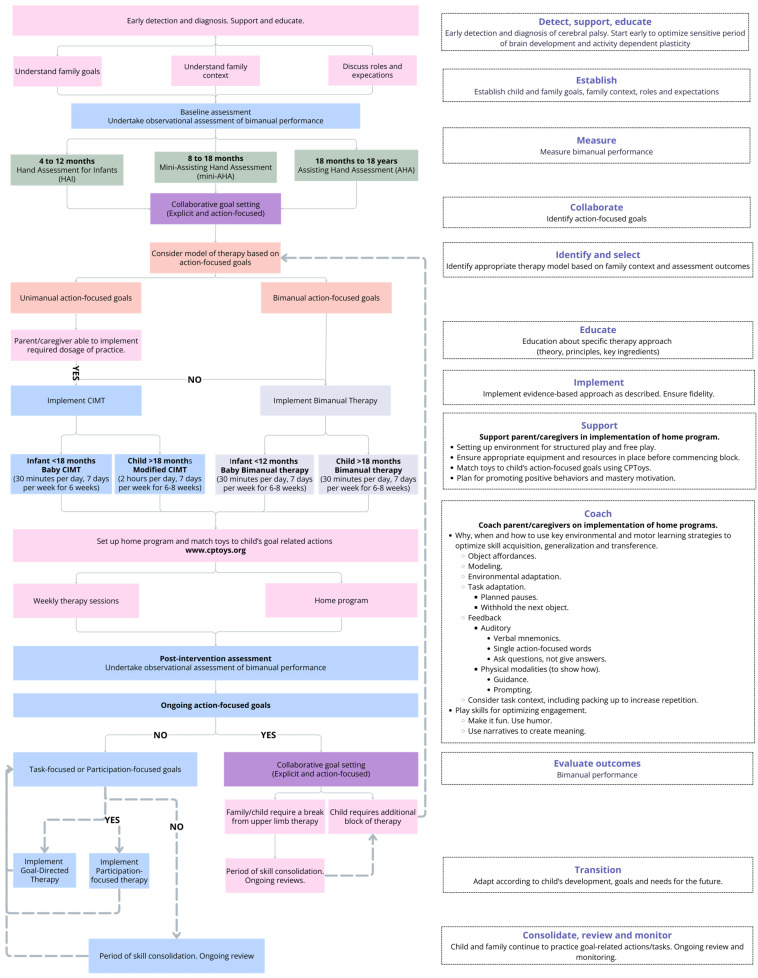
Proposed Model of Care for the implementation of evidence-based, distributed practice models of upper limb therapy.

Informed by research, evidence-based protocols, guidelines and frameworks for upper limb therapy, and the visual analysis of data from case series participants, and in consideration of the barriers and/or facilitators to the implementation of the upper limb therapy intervention protocols in this study, we have proposed a Model of Care (see Figure 3). This Model of Care outlines best practice care and service delivery for children with CP and their families as they progress from infancy to childhood and beyond. It starts with early detection and diagnosis; provides therapy approaches based on the highest levels of evidence; supports infants and children as individuals; educates, coaches, and supports families/caregivers; objectively evaluates outcomes; and aims to optimize skills development at the right time to progressively transition children away from ongoing activity-focused therapy so that they can spend time with their peers and families doing the things they want and need to do [50].

## 4. Discussion

This mixed-methods study, undertaken in a pragmatic clinical setting, demonstrated improved outcomes in bimanual performance following the implementation of multiple distributed models of upper limb therapies in a cohort of nine children with unilateral CP. The outcomes from this study make an important contribution to both research and clinical practice for infants and children with unilateral CP by not only reporting individual outcomes but also by examining the key considerations for the implementation of these approaches in a pragmatic clinical setting. Barriers to implementation included the complexity of the therapy approaches and cost. Facilitators included the evidence base and adaptability of the approaches that allowed clinicians to respond to an individual child and family’s needs.

Most children in this study commenced therapy under 12 months of age. During the first 2 years of life, therapy blocks occurred more frequently, with shorter break times between blocks. This meant children received more therapy during the period of most rapid gains in bimanual performance [3]. The model of upper limb therapy provided in each block was specific to each child. This was determined using valid and reliable assessments and consideration of family factors, such as determining the feasibility of a parent completing the home-based practice or parent acceptability or satisfaction with the perceived “fit” of the model of upper limb intervention indicated. This is an example of an important implementation strategy, which is to adapt and tailor (intervention) to context [43], with the goal of providing the right therapy at the right time. More than half of the children started with a single block of mCIMT, with the majority then receiving subsequent blocks of bimanual therapy. This reflects the application of a previously reported perspective that once a child improves unimanual actions and skills through mCIMT, they need to learn the cognitive, perceptual, and motor strategies to guide the performance of bimanual skills [13].

There was a wide range in the total number of blocks received by children (2–9 blocks), reflecting an adaptability to meet individual needs. This variation was not pre-determined and was influenced by several factors, including age at diagnosis and first access to therapy, baseline ability level, responsiveness to therapy, and family/social factors. The time between blocks was found to be shorter for the initial blocks (mean = 1.4 months) and increased for later blocks (mean = 3.1 months). This means infants and younger children received three to four blocks per year, while older children received two to three blocks per year. The average age at the transition from skills-based therapies (mCIMT, bimanual therapy) to GDT was 3 years, 3 months. This was also not predetermined but was guided by a child’s ability level and individual interest and motivation to perform self-care tasks independently. Importantly, for all children who transitioned to GDT, bimanual performance outcomes continued to improve or remained stable.

The outcomes suggest that children did not lose skills between blocks of therapy. This finding is important, as clinical experience suggests that many families of children with CP are concerned about a break in active therapy—even for only a few weeks. A feature of the distributed models of therapy that was identified as a facilitator of implementation is its design, where the time engaged in blocks of therapy can be clearly defined for families, providing the opportunity for a break between blocks to focus on other important goals for their child, e.g., gross motor or speech—or simply have a rest. The time between blocks also provided an important opportunity for the consolidation of goal-related actions and the ongoing practice of skills in spontaneous daily play routines [51].

In this study, all children had repeated assessments of their upper limb performance and showed a cumulative improvement in bimanual performance over time. For the children aged 18 months to 5 years who were assessed by the AHA, there was an average change of 14 AHA units. However, the rate of improvement and when the change occurred was highly variable. For example, of the six children who had repeated Mini-AHA assessments (between 8–18 months), three children showed rapid improvement from a single block during this early period (Figure 2). One child showed improvement after repeated blocks (Child 2). The two remaining children did not demonstrate early improvements but showed significant change (>5 AHA units) after 18 months of age. The reason for this variability is not yet fully understood but is likely to be related to factors such as the nature of a child’s brain injury, baseline ability level, cognition, the age at which a child started therapy, and family/social factors. Further research is required to better understand the impact of these variables on the development of bimanual performance. This highlights the complexity of delivering evidence-based upper limb therapies and the contextual factors that can impact both intervention and implementation outcomes [13].

Guided by research evidence, best practice guidelines and frameworks for children with CP [52,53], and the findings from this study, the proposed Model of Care (Figure 3) highlights key considerations for the delivery of an effective and evidence-based upper limb assessment and therapy service for children with unilateral CP. It incorporates a family-centered approach where therapists educate, coach, and support parents/caregivers to achieve important and meaningful goals for their child. Contemporary motor learning theories and effective strategy use underpin the models of therapy. The Model of Care is guided by principles of experience-dependent plasticity, where therapy is provided at the right time during the sensitive period of brain development when infants and children are most rapidly learning new actions and behaviors. Importantly, the Model of Care is adaptable and transitional. The aim is to provide the right therapy services at the right time, with a focus on reducing the need for ongoing activity-level therapy once a child and family start to prepare for the important transition to school and beyond.

Using the Updated Consolidated Framework for Implementation Research [39] and the Conceptual Model for Implementation Research (CFIR) [42], the retrospective review undertaken in this study identified barriers and facilitators to the effective implementation of evidence-based upper limb therapy approaches in clinical practice. Important facilitators of the therapy approaches used in the study include the strong evidence base and the adaptability to meet an individual child’s and their family’s needs, rather than a ‘one size fits all’ approach. However, we acknowledge the outcomes of this study need to be considered in the following context; services were provided by expert clinicians in a small specialist private practice specifically for children with CP; the clinicians were involved in the development of the therapy approaches—therefore, no training was required; both were certified raters or teachers of the assessment tools—no training was required; all services were government-funded, and there were no financial barriers to providing the blocks of therapy that were required. As a result, the implementation strategies [43] applied within different organizations, cultures, and service structures are likely to be different from those identified in this study. At an organizational level, these may include training and educating stakeholders, support for clinicians, changing/financing infrastructure such as equipment, and adapting how services are provided. Clinicians should be supported to receive training in the therapy approaches and the reliable administration of outcome measures as part of ongoing professional development opportunities. Other resources, such as toys and equipment, require funding and are essential for the successful implementation of these approaches. Further research is needed to evaluate additional implementation outcomes of the proposed Model of Care across different service systems.

### 4.1. Limitations

We acknowledge several limitations in this study due to the nature of a pragmatic, mixed-methods design evaluating clinical practice. There is no control group, and the effect of maturation and variability in patient characteristics, e.g., cognitive impairment, is unknown. The sample size is small; therefore, the results from the clinical outcomes cannot be generalized. Future research is required with a larger clinical cohort to examine trends in responsiveness over time and to strengthen support for the proposed Model of Care. The youngest age at which a child commenced services was 7 months, so the longitudinal effect of commencing therapy earlier is unknown and should also be investigated in future research. The timing of data collection was inconsistent and was administered by the child’s treating therapist, who was not blinded to the therapy received (BH, SG). We attempted to minimize potential scoring bias by coding each video file and undertaking scoring in a random order by a different, single assessor (ET). The review of implementation outcomes was completed by authors retrospectively, and there were no formal measures of implementation relating to feasibility or fidelity recorded during the study period. All these factors reflect the pragmatic nature of the study and limit the generalizability of the outcomes. Further research is needed to evaluate implementation and intervention effectiveness outcomes using the proposed Model of Care across different organization and service systems.

### 4.2. Practical Applications

The context for the implementation of the services provided in this study was a small Australian-based specialist pediatric private practice for children with CP. All services were provided within a clinic setting by highly experienced clinicians. Families/caregivers received federal government funding for these services, with no financial cost to them. Importantly, the resources required for this model of service delivery were lower than the resources required for the implementation of “usual care” models that have been found to be ineffective for children with CP [26]. Children received a maximum of three 8-week blocks of therapy, which equates to 24 sessions of therapy per year. Compared to fortnightly usual care models (26 sessions per year), the service outcomes for this study demonstrate no additional cost impact for the implementation of evidence-based upper limb therapy for children with CP. The service provided is sustainable and continues to be implemented with a growing team and a substantially larger number of clients in the context of Australia’s National Disability Insurance Scheme (NDIS) [54]. We believe the proposed Model of Care may also be possible to implement across most service settings in developed countries to maximize lifelong upper limb outcomes.

## Figures and Tables

**Table 1:** Participant characteristics, summary of therapy received, and change over time.

Child	Sex	MACS	More-Affected Side	Age at 1st Contact (Months)	First Block	Initial HAI Units	CIMT Total Blocks	BIM Total Blocks	Total Blocks	Initial Mini-AHA Units	Final Mini-AHA Units	Change Mini-AHA Units	Initial AHA Units	Final AHA Units	Change AHA Units	Transition to GDT
1	F	1	L	15	BIM	-	0	2	2	53	64	11	77	78	1	2 yrs 5 mths
2	F	1	L	7	BIM	7 months EaHS: R 24 EaHS: L 7 BoHM: 53	0	8	8	47	54	7	57	71	14	2 yrs 9 mths
3	M	2	R	12	CIMT	12 months EaHS: R 12 EaHS: L 24 BoHM: 60	1	8	9	47	60	13	54	67	13	4 yrs 2 mths
4	M	2	R	5	CIMT	10 months EaHS: R 24 EaHS: L 5 BoHM: 48	3	6	9	23	-	-	16	47	31	4 yrs 3 mths
5	M	2	R	9	CIMT	-	1	5	6	33	30	−3	47	55	8	*
6	M	2	L	6	CIMT	7 months EaHS: R 20 EaHS: L 4 BoHM: 42	1	7	8	-	-	-	46	54	8	3 yrs
7	F	2	R	7	CIMT	-	3	4	7	30	40	10	49	71	22	4 yrs 1 mth
8	M	2	R	33	BIM	-	0	3	3	-	-	-	54	65	11	4 yrs 1 mth
9	M	2	R	12	CIMT	-	1	6	7	53	56	3	52	71	19	4 yrs 4 mths

* Child 5 moved interstate before completing blocks of skills-based therapy. F: Female, M: Male, MACS: Manual Ability Classification System, L: Left, R: Right, BIM: Bimanual therapy, CIMT: constraint-induced movement therapy, HAI: Hand Assessment for Infants, EaHS: Each Hand Score, BoHM: Both Hands Measure, Mini-AHA: Mini-Assisting Hand Assessment, AHA: Assisting Hand Assessment, GDT: Goal-directed therapy, yrs: years, mths: months.

## Data Availability

Data are unavailable due to ethical restrictions.

## Supplementary Materials

The following supporting information can be downloaded at: https://www.mdpi.com/article/10.3390/jcm14030924/s1, Table S1: The TIDieR-Rehab checklist describing the distributed models of upper limb therapy implemented [55,56,57,58,59,60,61,62,63,64,65,66,67].
