# Supplementary material for: Barriers, Facilitators, and a Proposed Model of Care for Implementation of Upper Limb Distributed Practice Approaches for Children with Unilateral Cerebral Palsy
Source: J Clin Med. 2025 Jan 30;14(3):924. doi: 10.3390/jcm14030924 (PMC11818241; doi:10.3390/jcm14030924)
Supplement: Supplementary file 1 [file jcm-14-00924-s001.zip › jcm-3410687-supplementary.pdf]

Supplementary Table S1. The TIDieR- Rehab checklist describing the distributed models of upper limb therapy implemented.

TIDieR item	
1. Brief name	Distributed models of evidence-based upper limb therapy in infants and pre-school aged children with unilateral cerebral palsy (CP).
2. Why	**Rationale**<br>• Evidence-based upper limb therapy approaches provided early maximizes experience dependent neural plasticity [8,9] during the sensitive period of brain development.<br>• The most rapid gains in bimanual abilities occurs in the first 3 years of life [3,11,12].<br>• Implementation of time limited, distributed blocks of upper limb therapy are an effective way to deliver modified Constraint-induced movement therapy (mCIMT) and bimanual therapy [17-19].<br>• Intensive and distributed upper limb therapy approaches for children with unilateral CP over age 18 months are effective [25,26], however not all children respond to a single block of therapy [27].<br>• Repeated blocks of upper limb therapy has a cumulative effect in developing manual ability in infants [28-31].<br>• Limited longitudinal research exists about the effects of multiple blocks of evidence-based upper limb therapy approaches, if the benefits are one-off, short term, plateau over time or are incremental [32].<br>• There is no Model of Care for the implementation of multiple blocks of evidence-based distributed approaches of upper limb therapy in clinical practice.<br>**Essential elements**<br>• Assessment of bimanual abilities and performance using valid and reliable assessments including the Hand Assessment for Infants (HAI) [38], the Mini-Assisting Hand Assessment (Mini-AHA) [39] or the Assisting Hand Assessment (AHA) [40].<br>• Implementation of time limited, repeated blocks of evidence-based upper limb therapy models including mCIMT and bimanual therapy.<br>• Parent education, coaching and support [55].
3. Who	Infants and children with unilateral CP, aged 0 to 5 years.
4. When	The aim was to start therapy as soon as possible after early detection or diagnosis of CP. Age at first contact ranged from 6 to 33 months. Each infant/child received repeated blocks of therapy while under the age of 5 years.
5. What	**Materials**<br>Prior to commencing therapy, and during each block, parents received coaching and comprehensive education about their child's therapy program. This covered concepts such as functional hand use and hand role differentiation [13], models of evidence-based therapy [33], principles and strategies of motor learning theory [56], environmental enrichment [57] and behavior management [58].<br>Parents were supported to implement a home program between appointments using goal-oriented coaching [55]. This included guiding parents in setting up their home environment to facilitate fun, motivating play; defining specific goals for each week (typically 1 to 4 goals); using the CPToys platform (https://www.cptoys.org/) to provide an understanding of appropriate toys to provoke repetitive practice of the desired goal related actions, and using specific motor learning strategies to elicit repetitive goal related skills/action in structured play.<br>For children receiving CIMT, a restraint device on the less-affected upper limb and the provision of intensive practice to the more-affected upper limb was used. For older children the restraint device was a comfortable neoprene mitt which was customised

using neoprene material and sewn by the treating therapist. For infants receiving Baby CIMT the restraint device included a sock, or a clip closing the end of a long sleeve.

Procedures

A transitional model for delivering services has been implemented at CPTherapy since its inception in 2014. This model focuses on the development of upper limb actions and skills that facilitate functional hand use during infancy and preschool years using evidence-based models of upper limb therapy, with a transition to goal-directed training (GDT) and participation focused approaches in preparation for school and beyond. Each child attended the CPTherapy clinic for assessment and intervention with one of two pediatric occupational therapists (BH, SG) as part of standard clinical practice. The model of upper limb therapy implemented for each block comprised of either baby CIMT, modified CIMT or bimanual therapy. The model used was determined by the child's goals and age, consideration of the families social/contextual situation and the outcomes of an assessment undertaken using the HAI, Mini-AHA or AHA. The length, duration and frequency of therapy sessions and home program practice was determined by the model of upper limb therapy being implemented. Each child received repeated blocks of therapy while under the age of 5 years. The frequency of blocks provided, the amount of time between blocks and the timing for transition to goal-directed training, was determined by clinical need and in collaboration with the child and family.

The following procedures were implemented.

Detect, support, educate

- Early detection and diagnosis of cerebral palsy. Start early to optimize sensitive period of brain development and activity dependent plasticity

Establish

- Establish child and family goals, family context, roles and expectations

Measure

- Measure bimanual performance using either the HAI, the Mini-AHA or the AHA.

Collaborate

- Identify action-focused goals in the context of child and family goals.

Identify and select

- Identify the appropriate therapy model based on family context and assessment outcomes

Educate

- Education about specific therapy approach (theory, principles, key ingredients)

Implement

- Implement evidence-based approach as described. Ensure provider and participant adherence to protocols (fidelity).

Support parent/caregivers in implementation of home program (feasibility).

- Setting up the environment. for structured play and free play.
- Ensure appropriate equipment and resources in place before commencing block of therapy.
- Match toys to child's action-focused goals using CPToys.
- Plan for promoting positive behaviors and mastery motivation.

Coach parent/caregivers on implementation of home programs.

- Why, when and how to use key environmental and motor learning strategies to optimize skill acquisition, generalization and transference.
    - Object affordances.
    - Modelling.
    - Environmental adaptation.
    - Task adaptation.
        - Planned pauses.

        - Withhold the next object.
    - Feedback
        - Auditory
        - Verbal mnemonics.
        - Single action-focused words
        - Ask questions, not give answers.
    - Physical modalities (to show how).
        - Guidance.
        - Prompting.
    - Consider task context, including packing up to increase repetition.
- Play skills for optimizing engagement.
    - Make it fun. Use humor.
    - Use narratives to create meaning.

Evaluate outcomes

- Bimanual performance

Transition

- Adapt according to child's development, goals and needs for the future.

Consolidate, review and monitor

- Child and family continue to practice goal-related actions/tasks. Ongoing review and monitoring.

Parent education, coaching and support

Before and during blocks of therapy parents are given information about key components of the intervention including functional hand use and hand role differentiation [13], the different models of evidence-based therapy [33], principles and strategies of motor learning theory [56], environmental enrichment [57], and optimizing positive behavior [58]. Parents are supported to implement a home program between appointments using goal-oriented coaching [55]. This includes guiding parents in setting up their home environment to facilitate fun, motivating play; defining specific goals for each week (typically 1 to 4 goals); using the CPToys platform (https://www.cptoys.org/) to provide an understanding of each goal and appropriate toys to provoke repetitive practice of the desired goal related actions; and how to use specific motor learning strategies to elicit repetitive goal related skills/action in structured play.

Modified constraint-induced movement therapy

The models of Baby CIMT [31,59] and modified CIMT [17,58] have previously been described and demonstrated effectiveness. These incorporate the two fundamental components of CIMT: the use of a restraint device (mitt) on the less affected upper limb and the provision of intensive practice to the more affected upper limb [26,59]. For older children the restraint device was a comfortable neoprene mitt. For infants receiving Baby CIMT the restraint device included a sock, or a clip closing the end of a long sleeve. Both approaches were based on motor learning theory [56]. They focused on the repetitive practice of goal-related actions and skills with the more affected upper limb (e.g., grasp, release, hold and transport of objects), which were chosen after assessment and in consultation with the family. Unimanual toys were carefully selected to provide sufficient challenge and successful outcomes. In doing so, motor learning was facilitated by practicing skills and the experience of using the hand through intensive practice [13].

Bimanual therapy

The bimanual therapy approach has also been previously described and evaluated [17,58]. This approach is defined as "a process of learning bimanual hand skills through the repetitive use of carefully chosen, goal related, two-handed activities that provoke specific

	bimanual actions and behaviours" [13] (p. 52). Sessions focused on repetitive whole-task practice of challenging, motivating and purposeful bimanual activities (i.e., toys and games), carefully selected to facilitate acquisition and generalization of skills and actions. This bimanual therapy model is based on motor learning theory [56] and cognitive theories [60-62] that guide strategies to facilitate active problem solving and a mediated learning experience. In younger children (typically less than 15 months of age) perception-action theory [63] was used to inform intervention, whereby desired goal related actions were elicited primarily by the properties or affordances of the toy/object. For all children, extrinsic feedback was focused on the outcome of the task rather than specific movements. Key strategies included modelling, physical assistance (to show how), verbal and non-verbal cues and task/environmental adaptation, which were graded to ensure practice was always moderately challenging.<br><br>**Goal-directed training**<br>Goal-directed training (GDT) is an evidence-based model of therapy that focuses on a specific goal which is meaningful to the child and family [64]. Performance of the goal-related activity is observed, a plan is developed collaboratively, and the goal is practiced over a defined period. The therapist uses a range of strategies to support the practice and to guide discovery of the most effective way to perform the task independently. This often includes making adaptations to the task and the environment [65]. In this study, GDT was used with children > 3 years. The timing of transition from mCIMT or bimanual therapy to GDT in this model of service delivery was guided by the child's bimanual ability level and individual interest and motivation to perform self-care tasks independently [66].
6. Who provided	Two highly experienced pediatric occupational therapists (SG, BH) provided all assessment and interventions across the 5-year period of data collection as part of routine clinical practice. Both therapists have a PhD in Occupational Therapy and were involved in development and evaluation of some of the therapy approaches used in the study. Both therapists were certified raters for the HAI, Mini-AHA and AHA. They were also teachers involved in the certification courses.
7. How	Individual, face-to-face therapy sessions with the treating occupational therapist, parent and child present.
8. Where	A clinic setting at a CP-specific pediatric private practice in Melbourne, Victoria. Australia. In this setting, all therapy was undertaken with the infant or child sitting in an appropriate chair (for age and postural skill) with a height adjustable table positioned just below the forearm. The therapist sat in front of the child (for infants) or beside or behind the child. Parents were seated near the child but beyond the child's reach and were always active participants in the sessions.<br>The home program was undertaken in the parents/caregiver's home. Parents/caregiver were encouraged to undertake the structured play sessions with the infant or child sitting in an appropriate chair (for age and postural skill) with an appropriate tray or table that was positioned just below the infants/child's forearm.
9. How Much	**Baby-CIMT** approach (infants <18 months). Each intervention period was 6 weeks in length. Weekly 60-minute therapy sessions in the clinic were provided. Restraint wearing time was 30 min per day, 7 days per week. Application of restraint for daily home-based practice could occur over two shorter periods to maximize opportunities when the infant/child was alert, motivated and engaged to play and repetitively practice their unimanual goal-related actions and skills. Caregivers documented in a logbook the total period the mitt was worn per day, activities undertaken and any issues that arose.<br>**Modified CIMT** approach (children aged 18 months to 5 years). Each intervention period was 6 to 8 weeks in length. Weekly 60-minute therapy sessions in the clinic were provided. Restraint wearing time 2 hours per day, 7 days per week. Application of

	restraint for daily home-based practice could occur across short sessions (minimum 30-minute duration) to maximize opportunities when the infant/child was alert, motivated and engaged to play and repetitively practice their unimanual goal-related actions and skills. Caregivers documented in a logbook the total period the mitt was worn per day, activities undertaken and any issues that arose.<br>**Bimanual therapy** approach (children aged 0 to 5 years). Intervention period was 6 to 8 weeks in length. Weekly 60-minute therapy sessions in the clinic were provided. Daily home-based practice was encouraged for 30 minutes per day, 7 days per week. For children aged <18 months, daily practice of bimanual goal-related actions and skills could occur over two shorter periods to maximize opportunities for child being alert, motivated and engaged to play.<br>**Goal-directed therapy** approach (children aged 3 to 5 years). Intervention period for children who transitioned to GDT was approximately 2 weeks. A total of 2-4, 60-minute therapy sessions in the clinic were provided. Daily home-based practice of the goal-related task was encouraged. The total time was not specified. Instead, children were encouraged to practice until a specific number of successful trials were achieved.
10. How challenging	Consistent with the key principle of motor learning theory, all activities used in the structured play sessions were moderately challenging. Guided by action-perception theory [52] carefully selected toys were used in all therapy approaches. These toys were matched to a child's age and specific goals to provide a sufficient challenge, opportunities for learning and success. The treating therapists used a range of motor learning strategies to grade the challenge provided to the infant/child. These strategies have been outlined by Taghizedah et al. (2024) [67].
11. Regression/Progression	The dosage for each block of therapy was guided by the defined dosage parameters for the specific therapy approach provided (see "How much"). The progression of specific actions and skills were consistent with those outlined by Greaves and Hoare (2024) in Figures 1 and 2 [52].
12. Personalization	Intervention was individualized for each child based on clinical need, due to the pragmatic nature of this study. The model of upper limb therapy implemented for each block comprised of either baby CIMT, modified CIMT or bimanual therapy and was determined by the child's goals and age, consideration of the family's social/contextual situation and the outcomes of an assessment undertaken using the HAI, Mini-AHA or AHA.<br>Following assessment, if goals were unimanual in nature mCIMT was recommended. Bimanual therapy was recommended for children with bimanual goals [12,57]. Each child received repeated blocks while under the age of 5 years. The frequency of blocks provided, the amount of time between blocks and the timing for transition to GDT, was individually determined by clinical need and in collaboration with the child and family. Tailoring of intervention also occurred within individual therapy sessions through the application of motor learning strategies to enhance the child's skill acquisition [13]. Variations in the type of task, type of practice, and type of feedback were based on a child's age, cognition, ability level. The strategies used have been outlined by Taghizedah et al. (2024) [67].
13. Protocol deviations	None
14. How well	Both treating therapists in this study were involved in development and evaluation of the therapy approaches used in the study. As a result, intervention adherence or fidelity were not assessed.
15. Harms	In this pragmatic study, adverse events were monitored as part of routine clinical practice. There were no adverse events during the study period (2014 to 2020).
